# FHL2 deficiency impairs follicular development and fertility by attenuating EGF/EGFR/YAP signaling in ovarian granulosa cells

**DOI:** 10.1038/s41419-023-05759-3

**Published:** 2023-04-05

**Authors:** Chen Wang, Hui Sun, John S. Davis, Xiaojie Wang, Lijun Huo, Nan Sun, Qianzhi Huang, Xiangmin Lv, Cheng Wang, Chunbo He, Changjiu He, Yang Zhou, Jiyun Wu, Liguo Yang, Guohua Hua

**Affiliations:** 1 Key Lab of Agricultural Animal Genetics, Breeding and Reproduction of Ministry of Education, College of Animal Science and Technology, Huazhong Agricultural University, Wuhan, Hubei province 430070 China; 2 Olson Center for Women’s Health, Department of Obstetrics & Gynecology, University of Nebraska Medical Center, Omaha, NE 68198 USA; 3 VA Nebraska-Western Iowa Health Care System, Omaha, NE 68105 USA; 4 Vincent Center for Reproductive Biology, Vincent Department of Obstetrics and Gynecology, Massachusetts General Hospital, Harvard Medical School, Boston, MA 02114 USA; 5 National Center for International Research on Animal Genetics, Breeding and Reproduction (NCIRAGBR); Frontiers Science Center for Animal Breeding and Sustainable Production, Huazhong Agricultural University, Wuhan, 430070 PR China

**Keywords:** HIPPO signalling, Infertility

## Abstract

Female subfertility is an increasing reproductive issue worldwide, which is partially related to abnormal ovarian follicular development. Granulosa cells (GCs), by providing the necessary physical support and microenvironment for follicular development, play critical roles in maintaining female fertility. We previously showed that ectopic expression of four and a half LIM domains 2 (FHL2) promoted ovarian granulosa cell tumor progression. However, its function in follicular development and fertility remains unknown. Here, we confirmed that FHL2 is highly expressed in human and mouse ovaries. FHL2 immunosignals were predominantly expressed in ovarian GCs. A *Fhl2* knockout (KO) mouse model was generated to examine its roles in follicular development and fertility. Compared with wildtype, knockout of *Fhl2* significantly decreased female litter size and offspring number. Furthermore, *Fhl2* deficiency reduced ovarian size and impaired follicular development. RNA-sequencing analysis of GCs isolated from either KO or WT mice revealed that, *Fhl2* deletion impaired multiple biological functions and signaling pathways, such as *Ovarian Putative Early Atresia Granulosa Cell*, *ErbB*, *Hippo/YAP*, etc. In vitro studies confirmed that FHL2 silencing suppressed GCs growth and EGF-induced GCs proliferation, while its overexpression promoted GC proliferation and decreased apoptosis. Mechanistic studies indicated that FHL2, via forming complexes with transcriptional factors AP-1 or NF-κB, regulated *Egf* and *Egfr* expression, respectively. Besides, FHL2 depletion decreased YAP1 expression, especially the active form of YAP1 (nuclear YAP1) in GCs of growing follicles. EGF, serving as an autocrine/paracrine factor, not only induced FHL2 expression and nuclear accumulation, but also stimulated YAP1 expression and activation. Collectively, our study suggests that FHL2 interacts with EGFR and Hippo/YAP signaling to regulate follicular development and maintain fertility. This study illuminates a novel mechanism for follicular development and a potential therapeutic target to address subfertility.

## Introduction

Subfertility is a raising reproductive issue worldwide that affects one in six couples clinically, and half of them have no explanation for delaying pregnancy [1]. The number and the quality of follicles in ovary is highly related with a female’s fertility status [2]. Granulosa cells (GCs), which provide physical support and create microenvironments via secreting multiple growth factors and hormones, are critical to maintain follicular development and female physiology [3, 4]. Dysfunction of GCs results in abnormal follicular development and ovulation, and compromises female fertility [5]. Therefore, understanding the mechanisms underlying the regulation of GCs and follicular development is important for developing new strategies to eliminate infertility or subfertility associated with GC dysfunction.

Four and a half LIM domains 2 (FHL2) is a multifunctional protein that functions as an adaptor or scaffold to support the assembly of multi-protein complexes for critical cellular processes, including gene expression, cell survival, apoptosis, proliferation, differentiation, and other biological events [6]. FHL2 is expressed in the human and mouse ovaries [7–9]. We have shown that overexpression of FHL2 induces ovarian GC transformation and promotes granulosa cell tumor (GCT) progression [7]. Besides, FHL2 stimulates expression of inhibin subunit alpha (INHA) and cytochrome P450, cholesterol side chain cleavage (CYP11 A1), indicating that FHL2 is involved in the regulation of GC function [9]. However, the function and mechanism by which FHL2 regulates follicular development and fertility remain unknown.

In addition to gonadotropins such as follicle stimulating hormone (FSH) and luteinizing hormone (LH), intraovarian growth factors, by interacting with gonadotropin or other signaling pathways, play critical roles in follicular development, ovulation and fertility regulation [10–13]. For example, our recent study showed that, epidermal growth factor receptor (EGFR), which was rapidly co-activated by FSH, was involved in follicular growth regulation [14]. LH activates a cascade of signaling events to trigger ovulation. During the ovulatory response, LH induces expression of epidermal growth factor (EGF)-like growth factors and transactivation of EGFR, which are essential for ovulation [15, 16]. EGFR deficiency induces severe subfertility in a mouse model [17]. As a ligand of EGFR, epidermal growth factor (EGF), a growth factor secreted by ovarian GCs, could suppress the spontaneous onset of apoptosis and induce proliferation of GCs, follicular development and ovulation, by activating a downstream cellular signaling pathways such as PI3K/AKT, MAPK/ERK, Hippo/YAP, etc [7, 10, 18–20]. We reported that the major effector of Hippo signaling, Yes-Associated Protein 1 (YAP1), is spatiotemporally expressed in ovarian GCs and plays a critical role in the regulation of follicle development, ovarian physiology, and female fertility [21–23].

In the present study, we showed that FHL2 was highly expressed in ovarian tissue and GCs. FHL2 deficiency impaired follicular development and reduced fertility of female mice. RNA-sequencing analysis indicated *ErbB* and *Hippo/YAP* pathways are involved in *Fhl2* KO induced subfertility. Consistently, FHL2 deficiency downregulated EGF, EGFR and YAP1 expression both in vivo and in vitro cultured GCs. Besides, FHL2 depletion attenuated the active form of YAP1 in ovarian GCs of growing follicles. EGF treatment, in turn, promoted FHL2 expression and translocation from the cytoplasm to the nucleus, and stimulated YAP1 expression and activation. These data suggest that FHL2 played a critical role in regulating follicular development and female fertility by interacting with EGFR and Hippo/YAP signaling in ovarian GCs. Deficiency of FHL2 could induce subfertility in female mice.

## Results

### The expression and localization of FHL2 in the ovarian follicles

**Fig. 1:**
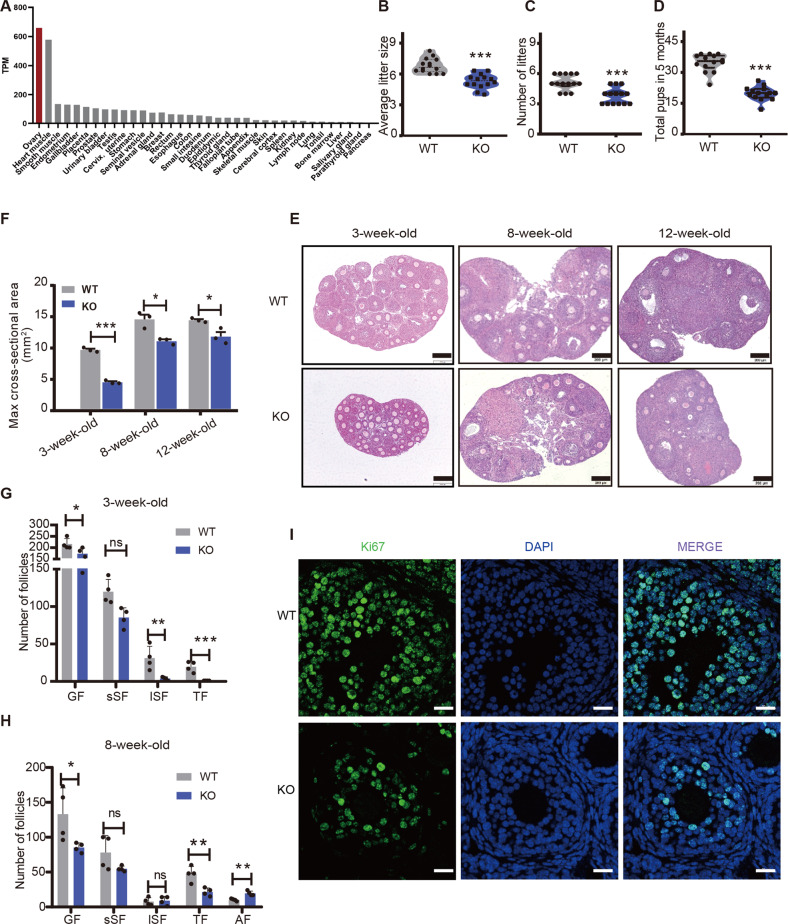
The expression and localization of FHL2 in the ovarian follicles. **A** The protein expression profiles of FHL2 in human tissues. Data credit: Human Protein Atlas. Data summary image is derived from the human protein atlas database (ps://v18.proteinatlas.org/ENSG00000115641-FHL2/tissue). **B**–**D** The Average litter size (**B**), the number of litters/female (**C**), and total pups (**D**) in wildtype (WT) and *Fhl2* knockout (KO) mice during 5-months breeding trial. *N* = 14 for each group. **E** Hematoxylin and eosin (HE) staining of ovaries isolated from 3-week-old, 8-week-old, and 12-week-old of WT and *Fhl2* KO mice. Scale bars: 200 μm. **F** Max cross-sectional area of ovaries from 3-week-old, 8-week-old, and 12-week-old of WT and *Fhl2* KO mice. *N* = 3 for each group. **G**, **H** Number of follicles per ovary isolated from 3-week-old WT or KO mice (**G**), and 8-week-old WT or KO mice (**H**). *N* = 4 for each group. GF total growing follicle, sSF small secondary follicle (wrapped up by 2–5 layers of granule cells), lSF large secondary follicles (wrapped up by more than 5 layers of granule cells), TF tertiary Follicles, AF atretic follicle. **I** Representative image of immunofluorescence staining of Ki67 in WT and *Fhl2* KO mice ovaries. Green color indicated Ki67 immunosignal. Nuclei were staining with DAPI in blue. Scale bar: 20 µm. Student’s *t* test was used to compare the difference between groups. Data are shown as mean ± SEM. *P < 0.05, **P < 0.01, ***P < 0.001. ns non-significant.

Our previous study showed that FHL2 is highly expressed in human ovarian granulosa cell tumors and epithelial tumor tissues and cells [7, 24]. To identify FHL2 expression patterns in the normal tissues, 36 different tissue profiles were extracted from the human protein atlas database. The results showed that ovarian tissue exhibited the highest FHL2 expression, followed by the heart muscle. The expression levels of FHL2 in other tissues are much lower than that in the ovary (Fig. 1A). Immunohistochemistry staining in mouse ovary confirmed that FHL2 was predominantly located in GCs of the growing follicles, including primary, secondary, and antral follicles (Fig. S1). Immunofluorescence analysis in vitro cultured primary GCs further indicated that FHL2 was co-localized with F-actin fibers and condensed as punctuating structures at the end of the fibers (Fig. S2, upper panel). And the immunosignal was enriched in the nuclei of dividing cells compared to that of the non-dividing cells (Fig. S2, lower panel), indicating that FHL2 expression and localization may be related to cell cycle control. Western blot analysis of cytoplasmic and nuclear extracts of GCs confirmed FHL2 expression both in the cytoplasm and nucleus (Fig. S3).

### Deletion of *Fhl2* impaired follicular development and reduced female fertility

To further understand the role of FHL2 in follicular development and fertility, we constructed a universal *Fhl2* knockout mouse model using the CRISPR/Cas9 system by deleting exons 3–5 (Fig. S4A). Genotyping by PCR, sequencing, and western blot results confirmed the successful deletion of *Fhl2* in mouse ovaries (Fig. S4B–E). After mating with fertility-certified male mice, we found that the average litter size of KO mice decreased significantly compared with WT (Fig. 1B). Besides, the litters per *Fhl2* KO female mice delivered in the tested period were significantly lower than that observed in the WT mice (Fig. 1C). In total, the *Fhl2* KO mice gave birth to 40% fewer pups than WT mice during the breeding trial (Fig. 1D).

Morphologic and histologic analyses of ovaries from 3-, 8- and 12-week-old age-matched WT and KO mice showed that, *Fhl2* deletion reduced ovarian size dramatically (Fig. 1E, F, Fig. S5). The 3-week-old WT mice exhibited different stages of growing follicles in ovaries, including early secondary (2–5 layers of GCs per follicle), large secondary (over 5 layers of GCs per follicle), and tertiary (antral) follicles. In contrast, the ovaries of age-matched KO mice showed mainly early secondary follicles, and few large secondary or antral follicles (Fig. 1G, Fig. S6). Serial follicle counting further illustrated that *Fhl2* deletion significantly decreased the number of total growing follicles, large secondary and tertiary (antral) follicle numbers of pre-pubertal mice (3-week-old) when compared with WT mice (Fig. 1G, Fig. S7). Data from sexual mature mice (8-week-old) confirmed that *Fhl2* deletion decreased the total growing follicles and tertiary (antral) follicles, increased follicular atresia when compared with WT mice (Fig. 1H, Fig. S7). Immunofluorescence staining confirmed that, the immunosignals of cell proliferation marker Ki67 were more abundant in GCs of WT mice as compared that of KO mice (Fig. 1I). Collectively, these findings indicate that *Fhl2* deletion impaired female fertility and follicular development.

### *Fhl2* knockout impaired pathways related with follicular development

**Fig. 2:**
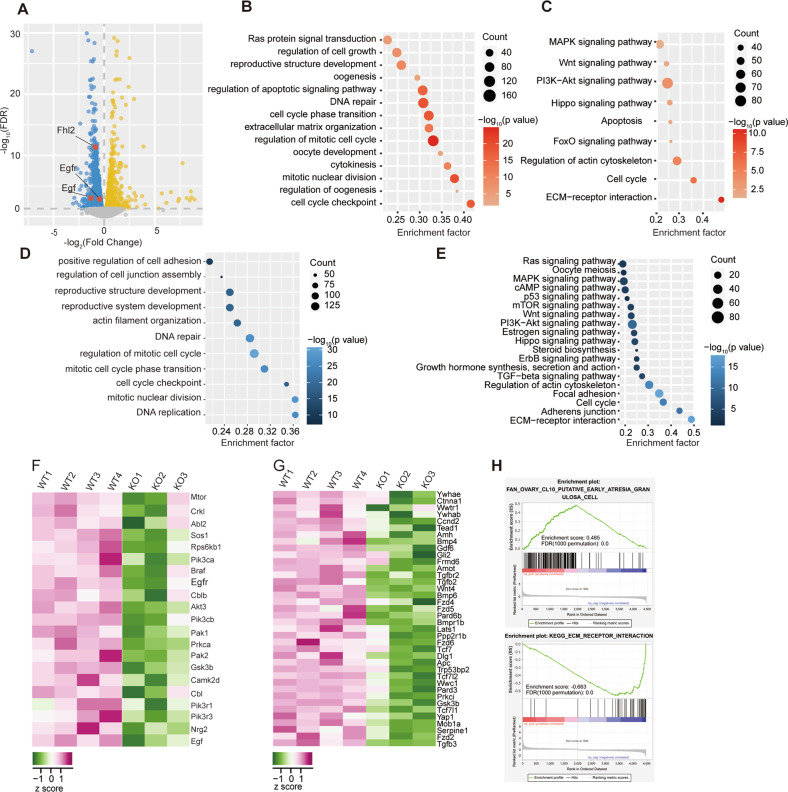
Bioinformatics analysis of differentially expressed genes in ovarian GCs of 3-week-old WT and *Fhl2* KO mice. **A** Volcano plot showing differentially expressed genes (DEGs) (FDR ≤ 0.05) between WT and *Fhl2* KO mice. Yellow and blue dots represent the up-regulated or down-regulated genes in KO mice, respectively. *Fhl2*, *Egfr* and *Egf* were indicated by red dots. **B**, **C** Representative GO and KEGG enrichment results (*P* ≤ 0.05) of all DEGs between ovarian GCs of WT and KO mice, respectively. **D**, **E** Representative GO and KEGG enrichment results (*P* ≤ 0.05) of down-regulated DEGs in *Fhl2* KO mice ovarian GCs. **F** Expression heatmap of DEGs in *Erbb* signaling pathway. **G** Expression heatmap of DEGs in *Hippo* signaling pathway. **H** Representative Gene Set Enrichment Analysis (GSEA) results (FDR ≤ 0.05) of all DEGs between WT and KO mice ovarian GCs. The FDR is obtained from the *P*-value based on 1000 times permutation test.

To determine the regulatory roles of FHL2 in follicular development, we conducted RNA-sequencing of the primary GCs isolated from either WT or KO mice ovaries. A total of 4803 genes were differentially expressed (FDR ≤ 0.05) in *Fhl2* KO GCs compared with WT transcriptome (Fig. 2A). GO analysis of all differentially expressed genes (DEGs) enriched biological processes such as *oogenesis, DNA repair, mitotic nuclear division, regulation of cell growth, and cytokinesis*, *etc* (Fig. 2B). KEGG analysis revealed pathways altered by *Fhl2* knockout, including *ECM-receptor interaction, cell cycle, Apoptosis, PI3K-AKT*, *Hippo*, *FoxO*, *etc* (Fig. 2C). Further analysis of downregulated DEGs in KO mice enriched the biological progresses of *reproductive system development*, *DNA replication, regulation of mitotic cell cycle, etc* (Fig. 2D). And KEGG pathway analysis revealed that these downregulated DEGs were mainly enriched in signaling pathways such as *Ras*, *ErbB, MAPK*, *PI3K-AKT*, *Hippo*, *cAMP, etc* (Fig. 2E). Heatmap of *Hippo* and *ErbB signaling pathways* displayed that multiple molecules were downregulated in GCs of *Fhl2* KO mice, including *Egf*, *Egfr* and *Yap1* (Fig. 2F, G). Gene set enrichment analysis (GSEA) revealed that *Fhl2* deletion was negatively correlated with pathways such as *Extracellular Matrix (ECM) Receptor Interaction* (Enrichment score = −0.663, FDR = 0), *Integrin1 pathway* (Enrichment score = −0.714, FDR = 0) (Fig. 2H, Fig. S8). GSEA also showed that *Fhl2* deficiency was positively correlated with *Ovarian Putative Early Atresia Granulosa Cell* (Enrichment score = 0.485, FDR = 0) (Fig. 2H), which is consistent with the phenotype of decreased growing follicles and increased atretic follicles in KO mice. Collectively, bioinformatics analysis data confirmed that follicular development related pathways were disrupted in *Fhl2* KO mice.

### FHL2 deficiency inhibited GC viability and EGF-induced GC proliferation in vitro

**Fig. 3:**
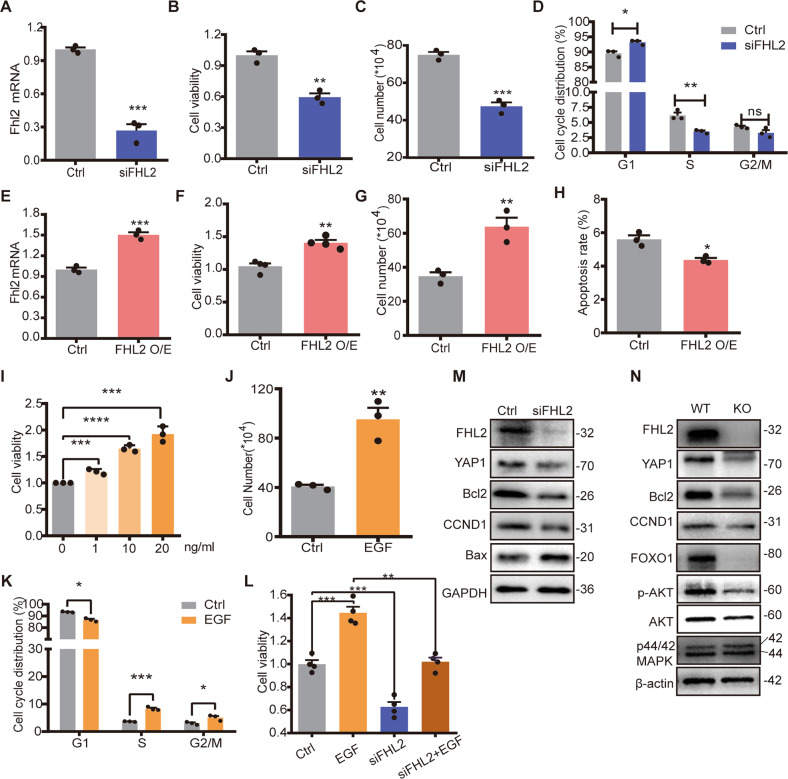
FHL2 deficiency inhibited GC viability and EGF-induced GC proliferation in vitro. **A** *Fhl2* mRNA levels in GCs transfected with scramble siRNA (Ctrl) or *Fhl2* specific siRNA (siFHL2). *N* = 3 for each group. β-actin was used as a loading control. **B** Knockdown of FHL2 inhibited cell viability significantly. Cell viability was detected by CCK-8. *N* = 3 for each group. **C** Quantitative data showing cell number changes in control and FHL2 knockdown cells. *N* = 3 for each group. **D** Flow cytometry showing cell cycle distribution in GCs transfected with scramble siRNA (Ctrl) or *Fhl2* specific siRNA (siFHL2). Data was displayed as a percentage of cells in each phase of the cell cycle. *N* = 3 for each group. **E** *Fhl2* mRNA levels in GCs transfected with control vector (Ctrl) or *Fhl2* overexpression vector (*Fhl2* O/E). *N* = 3 for each group. β-actin was used as loading control. **F** Overexpression of FHL2 promoted GC viability. *N* = 4 for each group. **G** Cell growth changes in control and FHL2 overexpression cells. *N* = 3 for each group. **H** Overexpression of FHL2 dramatically deceased cell apoptosis. Cells were stained with an Annexin V-APC/PI dual staining kit and apoptosis was analyzed by flow cytometry. *N* = 3 for each group. **I** EGF treatment improved cell viability in a dose-dependent manner. GCs were incubated in medium containing DMEM/F12 (1:1) and 1% fetal bovine serum (FBS) in the absence (vehicle control, Ctrl) or presence of 1, 10, or 20 ng/ml EGF for 48 h. CCK-8 was employed to measure GC viability. *N* = 3 for each group. **J** EGF treatment stimulated GC proliferation. GCs were incubated in the absence (vehicle control, Ctrl) or presence of EGF (20 ng/ml) in medium containing DMEM/F12 (1:1) and 1% FBS for 48 h. *N* = 3 for each group. **K** Cell cycle distribution in GCs treated with absence or presence of EGF. *N* = 3 for each group. Cell cycle analysis was performed by flow cytometry. **L** Knockdown of FHL2 attenuated EGF induced cell proliferation. GCs were transfected with scramble siRNA (Ctrl) or *Fhl2* specific siRNA (siFHL2) for 24 h, followed by EGF treatment (20 ng/ml) for 48 h. Cell viability was measured by CCK-8. *N* = 4 for each group. **M** Knockdown of FHL2 in vitro decreased protein levels related with cell growth. GCs were transfected with either scramble siRNA (Ctrl) or *Fhl2* specific siRNA (siFHL2) for 72 h. Protein levels were determined by western blot. GAPDH was used as a loading control. All the experiments were repeated at less 3 times and representative images were presented. **N** Representative western blot images showing the protein expressions in wildtype (WT) and *Fhl2* KO mice ovaries. Whole ovary extracts were prepared for western blot. β-actin was used as the loading control. All the experiments were repeated at less 3 times. Student’s *t* test or one-way ANOVA were used to compare the difference between groups. Data are shown as mean ± SEM. *P < 0.05, **P < 0.01, ***P < 0.001, ****P < 0.0001, ns non-significant.

GCs play important roles in follicular development and normal function maintenance. A large portion of proliferating GCs support the progression of follicular growth and maturation [14]. To further confirm FHL2 regulatory effect on GC growth, we conducted RNAi in vitro cultured primary GCs. Compared with GCs transfected with control siRNA (Ctrl), *Fhl2* specific siRNA transfection significantly reduced *Fhl2* expression (Fig. 3A). Knockdown of FHL2 significantly suppressed cell viability, reduced cell numbers, and arrested the cell cycle progression when compared with control (Fig. 3B–D). In contrast, transfection of GCs with a FHL2 overexpression vector (FHL2 O/E) increased *Fhl2* level, promoted cell proliferation, increased cell numbers, and decreased cell apoptosis compared with cells transfected with control vector (Ctrl) (Fig. 3E–H). These data suggest that FHL2 regulates GC growth.

EGF, as a core ligand for *ErbB* signal pathway, can effectively promote the proliferation of GCs and inhibit the follicle atresia [25]. We confirmed here that, EGF treatment promoted GC proliferation in a dose-dependent manner (Fig. 3I). Besides, EGF treatment significantly increased cell number and promoted cell cycle progression (Fig. 3J, K). Interestingly, knockdown of FHL2 abrogated EGF-induced cell proliferation (Fig. 3L). Connected with the finding that *Fhl2* deletion impaired *ErbB* signaling pathway (Fig. 2F), these data suggest that FHL2 is involved in EGF induced GC proliferation.

We then detected proliferation- and apoptosis-related protein expression in vitro cultured GCs transfected with FHL2 siRNA. Western blot results showed that the proliferation marker CCND1 and the anti-apoptosis related protein Bcl2 were downregulated, while the apoptosis-promoting protein Bax was up-regulated in FHL2 knockdown cells when compared with control (Fig. 3M, Fig. S9A). Consistently, western blot of whole ovary extracts of either WT or KO mice showed that follicle growth or GC proliferation related proteins CCND1, FOXO1, and anti-apoptosis Bcl2 were significantly decreased in *Fhl2* KO mice (Fig. 3N, Fig. S9B). Besides, AKT was downregulated in the KO mice extracts, indicating that PI3K/AKT signaling pathway was inhibited. Consistently, the immunosignals of the apoptosis marker cleaved-Caspase 3 was more abundant in growing follicles of KO mice compared with WT ovaries (Fig. S10). The levels of p44/42 MAPK remained unchanged between the KO and WT mice (Fig. 3N).

### FHL2 regulated *Egf* and *Egfr* transcription by binding with transcriptional factor AP-1 and NF-κB

**Fig. 4:**
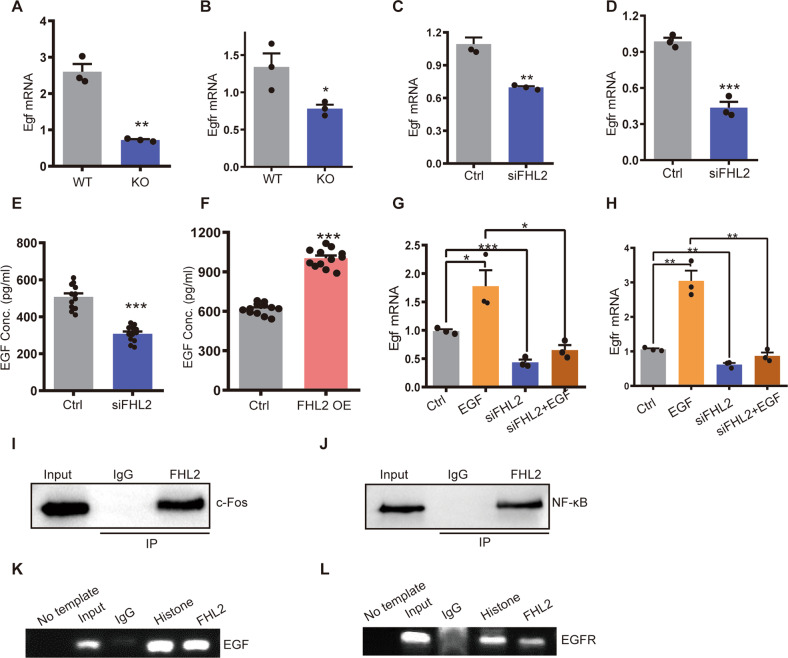
FHL2 regulated *Egf* and *Egfr* transcription by binding with transcriptional factor AP-1 and NF-κB. **A**, **B** QRT-PCR showing the *Egf* and *Egfr* mRNA levels of the GCs collected from 3-week-old WT and *Fhl2* KO mice. β-actin was used as loading control. *N* = 3 for each group. **C**, **D** QRT-PCR showing the *Egf* and *Egfr* mRNA levels of the GCs transfected with scramble siRNA (Ctrl) or *Fhl2* specific siRNA (siFHL2). β-actin was used as loading control. *N* = 3 for each group. **E** Knockdown of FHL2 reduced EGF concentration in the culture medium. GCs were transfected with scramble siRNA (Ctrl) or *Fhl2* specific siRNA (siFHL2) for 72 h. Culture medium were collected from each group and EGF concentration was measured by ELISA. *N* = 12 for each group. **F** Overexpress of FHL2 increased EGF concentration in the culture medium. GCs were transfected with control vector (Ctrl) or *Fhl2* overexpression vector (*Fhl2* OE) for 72 h. Culture medium were collected from each group and EGF concentration was measured by ELISA. *N* = 12 for each group. **G**, **H** Knockdown of FHL2 inhibited EGF induced gene expressions. GCs were transfected with either scramble siRNA (Ctrl) or *Fhl2* specific siRNA (siFHL2) for 24 h, followed by EGF treatment (20 ng/ml) for 48 h. QRT-PCR were performed to detect *Egf* and *Egfr* mRNA expression levels of each group. β-actin was used as a loading control. *N* = 3 for each group. **I**, **J** Co-immunoprecipitation assay showing the interaction of FHL2 with c-fos and NF-κB. **K**, **L** Chromatin immunoprecipitation (ChIP) assay showing that *Egf* and *Egfr* was the direct target of FHL2. Acetyl Histone H3 was used as a positive control, while samples from IgG group (antibody replaced with same amount of IgG) was used as a negative control. Student’s *t* test or one-way ANOVA were used to compare the difference between groups. Data are shown as mean ± SEM. *P < 0.05, **P < 0.01, ***P < 0.001.

RNA-sequencing data showed that *Fhl2* deletion downregulated core components of *ErbB* signaling pathway such as *Egf* and *Egfr* expression (Fig. 2). Quantitative RT-PCR data confirmed that *Egf* and *Egfr* were downregulated in GCs of KO mice compared that of WT (Fig. 4A, B). Knockdown of FHL2 in vitro cultured GCs also decreased *Egf* and *Egfr* mRNA levels (Fig. 4C, D). ELISA data further confirmed that EGF concentration was significantly decreased in the culture medium of FHL2 knockdown cells (Fig. 4E). In contrast, FHL2 overexpression increased EGF secretion in the culture medium (Fig. 4F). EGF acts as an autocrine/paracrine growth factor to drive follicular development. We observed that EGF treatment induced expression of *Egf* and *Egfr* in GCs. Interestingly, knockdown of FHL2 not only decreased basal level, but also blocked EGF-induced *Egf* and *Egfr* expression (Fig. 4G, H). Together with the finding that FHL2 deficiency inhibited EGF induced GC proliferation (Fig. 3L), these data indicated that FHL2 is required in the EGF/EGFR autocrine/paracrine loop to regulate GC growth.

FHL2 can bind with transcription factors to regulate gene expression in a cell/tissue specific-manner [7]. Bioinformatics analysis of the proximal promoter sequences of *Egf* and *Egfr* indicated that there are AP1-binding sites in the *Egf* promoter and NF-κB-binding sites in the *Egfr* promoter regions. Co-immunoprecipitation assays (Co-IP) were then applied to determine whether FHL2 interacts with AP-1 and/or NF-κB transcription factors in GCs. The presence of c-fos and NF-κB in FHL2 immunoprecipitates indicated that FHL2 interacts with AP-1 and NF-κB transcription factors in GCs (Fig. 4I, J). Finally, chromatin-precipitation (ChIP) assays convincingly indicated that FHL2/AP-1 complexes and FHL2/NF-κB complexes could bind to the *Egf* and *Egfr* promoters, respectively (Fig. 4K, L). These results indicate that FHL2 may act as a transcriptional co-factor to regulate *Egf* and *Egfr* transcription.

### FHL2 deficiency downregulates YAP1 and induces YAP1 cytoplasm subcellular location

**Fig. 5:**
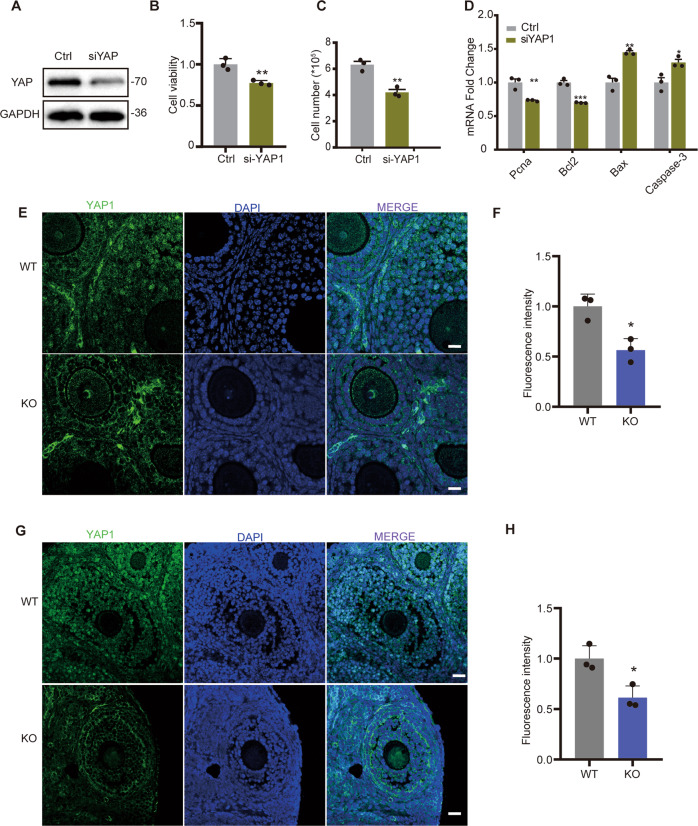
YAP1 depletion inhibited GC growth and FHL2 deficiency induced YAP signal translocation. **A** Representative western blot image showing YAP1 protein level. GCs were transfected with scramble siRNA (Ctrl) or *Yap1* specific siRNA (siYAP) for 72 h. YAP1 expression was detected by western blot. GAPDH was used as a loading control. Quantitative data showing YAP1 knockdown inhibited cell viability (**B**), and reduced cell number (**C**), *N* = 3 for each group. **D** Knockdown of YAP1 decreased gene expression related with cell proliferation. GCs were transfected with scramble siRNA (Ctrl) or *Yap1* specific siRNA (siYAP) and QRT-PCR were used to detect relative gene expressions. *N* = 3 for each group. **E** Immunofluorescence staining showing the expression and localization of YAP1 immunosignal in GCs of 3-week-old WT and *Fhl2* KO mice. Ovaries were isolated from 3-week-old WT and *Fhl2* KO mice and then fixed to perform immunofluorescence staining. Green color represents YAP1 immunosignal. Nuclei were stained with DAPI (blue). Scale bar: 20 µm. **F** Quantification of YAP1 immunosignal intensity in 3-week-old WT and *Fhl2* KO mice. Image J was used to quantify immunosignal intensity. *N* = 3 for each group. **G** Immunofluorescence staining showing the expression and localization of YAP1 immunosignal in 8-week-old WT and *Fhl2* KO mice GCs.Scale bar: 20 µm. **H** Quantification of YAP1 immunosignals in 8-week-old WT and *Fhl2* KO mice. *N* = 3 for each group. Student’s *t* test were used to compare the difference between groups. Data are shown as mean ± SEM. *P < 0.05, **P < 0.01, ***P < 0.001.

KEGG analysis indicated that *Fhl2* deficiency altered the *Hippo/YAP* pathway (Fig. 2E). YAP1 expression was significantly downregulated in *Fhl2* KO mice (Figs. 2G, 3N). Knockdown of *Fhl2* in vitro cultured GCs also decreased YAP1 expression (Fig. 3M). Our previous study showed that GC specific *Yap1* KO mice (Foxl2-CRE;Yap1^flox/flox^) displayed reduced ovarian size, increased GC apoptosis, and subfertility [23], similar to phenotype as observed in the *Fhl2* KO mice. Here we showed that, knockdown of YAP1 in vitro cultured GCs inhibited cell viability and reduced cell numbers compared with control group (Fig. 5A–C). QRT-PCR results demonstrated that knockdown of YAP1 dramatically suppressed expression of *Pcna* and *Bcl2*, and in parallel, increased *Bax* and *Caspases-3* (*Casp3*) expression (Fig. 5D). Together with data shown in Fig. 1 and Fig. 3, these data suggested that *Yap1* or *Fhl2* depletion induced similar phenotype in GCs growth and follicle development.

Our previous study showed YAP1 nuclear location in GCs is critical for driving follicular growth, and pharmacological inhibition of YAP1 activity disrupted mice ovarian follicle development [23]. Immunofluorescence results confirmed that YAP1 immunosignals intensity was significantly lower in GCs of pre-pubertal *Fhl2* KO mice ovaries compared with age-matched WT mice (Fig. 5E, F). Most importantly, robust YAP1 immunosignals were located in nuclei of GCs in growing follicles in WT ovaries, however, YAP1 immunosignals were mainly cytoplasmic in GCs of *Fhl2* KO mice ovaries (Fig. 5E). The difference of YAP1 expression and subcellular location was also observed in sexually mature mouse ovaries (8-week-old). YAP1 immunofluorescence intensity was higher in the WT mice ovary, where YAP1 immunosignals were dominantly identified in the nuclei of GCs. However, the KO mice showed lower YAP1 intensity and more cytoplasm location in GCs (Fig. 5G, H). These data indicated that FHL2 deficiency not only decreases YAP1 expression, but also induces its cytoplasm translocation (inactive form of YAP1).

### EGF treatment induces FHL2 and YAP1 expression in GCs

**Fig. 6:**
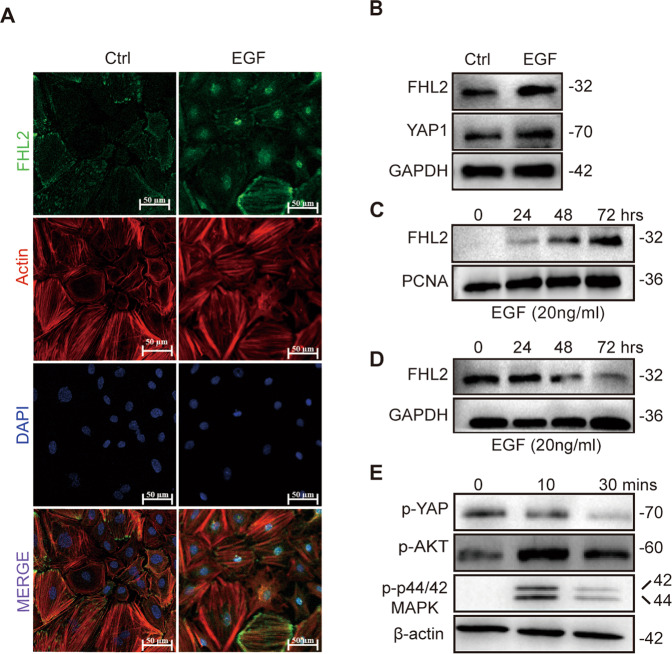
EGF Treatment Induced FHL2 and YAP1 Expression and Translocation. **A** Immunofluorescence staining showing FHL2 expression and subcellular location in vitro. Primary GCs were isolated and cultured with absence (vehicle control, Ctrl) or presence of 20 ng/ml EGF (EGF) for 48 h. Immunofluorescence staining was used to identify the subcellular location of FHL2. The FHL2 immunosignal was presented in green and F-actin (phalloidin) was presented in red color. Nuclei were stained with DAPI (blue). Scale bar: 50 μm. **B** FHL2 and YAP1 protein expression in GCs treated in absence (vehicle control, Ctrl) or presence of EGF (20 ng/ml). GAPDH was used as a loading control. **C**, **D** Western blot images showing FHL2 expressions in the nucleus (**C**) or cytoplasm (**D**) of GCs. GCs were incubated with absence (vehicle control, Ctrl) or presence of EGF (20 ng/ml) for 24 h, 48 h or 72 h in vitro. Nuclear and cytoplasmic protein were isolated to analyze FHL2 expression by western blot. PCNA was used as a nuclear protein loading control. GAPDH was used as a cytosol protein loading control. **E** EGF treatment stimulated the active form of YAP1. GCs were incubated with 20 ng/ml EGF for 0, 10 or 30 min. Phosphorylated protein levels were determined using western blot. β-actin was used as a loading control.

Translocation from the cytoplasm to nucleus will promote FHL2 binding to transcription factors, leading to activation of FHL2 target genes [26]. Immunofluorescence analyses showed that under serum starvation conditions, FHL2 was co-localized to the F-actin and focal adhesion complex in about 80–90% of GCs, with relatively weak signals in the nuclei (Fig. 6A, left panel). EGF stimulation dramatically induced translocation of FHL2 into the nuclei in over 90% of cells (Fig. 6A, right panel). In addition, long-term EGF treatment significantly induced FHL2 expression (Fig. 6B). Western blot of nuclear extracts of GCs indicated that EGF induced FHL2 accumulation in a time-dependent manner (Fig. 6C). Interestingly, FHL2 levels in the cytoplasmic fraction was decreased during long-term EGF treatment (Fig. 6D). These data confirmed that EGF could elevate FHL2 expression and trigger its nuclei accumulation.

EGF is also a well-identified upstream activator of YAP1 [23, 27]. Long-term EGF treatment stimulated YAP1 expression in GCs (Fig. 6B). Besides, treatment with EGF rapidly activated PI3K/AKT and MAPK/ERK and dephosphorylated YAP1, which indicate activation of YAP1 (Fig. 6E). Together with the facts that *Fhl2* KO mice displayed altered *Hippo/YAP signaling* (Fig. 2G), attenuated YAP1 expression (Fig. 3N), and induced cytoplasm translocation of YAP immunosignal (Fig. 5E–H), these results suggested FHL2 may regulate YAP1 expression and activation by regulating EGF/EGFR signaling.

## Discussion

Subfertility, including follicular development and ovulation disorders, is an increasing concern worldwide [28]. We previously reported that, ectopic expression of FHL2 initiated and promoted gynecologic carcinogenesis [7, 24]. The current study showed that *Fhl2* knockout mice displayed impaired follicular growth and were subfertile. Further research showed that *Fhl2* deficiency suppressed EGF and EGFR expression. Accordingly, *Fhl2* deficiency also decreased YAP1 expression, especially the active form of YAP1 (nuclear YAP1) in proliferating GCs. Collectively, our study suggests that FHL2 plays important roles in regulating follicular development to maintain fertility by interacting with EGF/EGFR and Hippo/YAP signaling pathway.

Although still fertile, *Fhl2* knockout mice had smaller litter size and less total offspring in a breeding trial. Morphologic and histologic analyses clearly showed that *Fhl2* deletion reduced ovarian size and total growing follicles, especially antral follicles in the ovary, and increased follicular atresia. Consistently, silico analysis suggested that *Fhl2* deletion impaired many biological processes in GCs such as *regulation of cell growth, cell cycle phase transition, DNA replication, regulation of apoptotic signaling pathway*, *etc*, and signaling pathways such as *ErbB*, *PI3K-AKT*, *Hippo*, *FoxO*, *Apoptosis*. Many of these processes and pathways were highly related with GC growth and follicular development. By employing primary GC as in vitro model, we confirmed that FHL2 knockdown decreased GCs proliferation ability and increased cell apoptosis. These data indicated that *Fhl2* deficiency induced subfertility might be related to GC dysfunction and compromised follicular development.

Our very recent published paper reviewed that the highly conserved Hippo pathway, especially its downstream effector YAP1, plays a critical role in governing ovarian physiology, fertility, and pathology [22]. Similar with the subfertile phenotype observed in *Fhl2* KO mice, our previous study proved that *Foxl2* promoter-driven knockout of *Yap1* in ovarian GCs also increased GCs apoptosis, disrupted follicular development and impaired fertility [23]. Besides, our current and previous study confirmed that inhibition or downregulation of YAP1 blocked GCs proliferation in vitro in several species, including human, bovine and mice [29–31]. Consistently, FHL2 knockdown also inhibited growth of both human and murine GCs [7]. Besides, we found that FHL2 depletion not only downregulated YAP1 expression, but also attenuated nuclear YAP1 in growing follicles. Collectively, these data indicate that FHL2 may interact with Hippo/YAP signaling to regulate follicular development and fertility.

Bioinformatics analysis revealed that *ErbB* signaling pathway was inhibited in GCs of *Fhl2* KO mice. EGF and EGFR, two core components of *ErbB* signaling pathway, are important in regulating follicle development and female fertility [32, 33]. Disruption of *Egfr* led to female infertility due to failed folliculogenesis in a zebrafish model [34]; and mice lacking *Egfr* expression were severely subfertile [17]. Despite their importance in follicular development, little is known about the transcriptional regulation of EGF and EGFR in ovarian cells. Sun et al claimed that FHL2 expression was positively correlated with EGFR expression in glioblastoma samples from patients and interacted with EGFR in glioblastoma cells [35]. Our previous study also showed that FHL2 expression level was correlated with EGFR expression in the human granulosa cell tumor cell lines KGN and COV434 [36]. Here, we showed that FHL2 depletion downregulated EGF and EGFR expression both in vivo and in vitro. Multiple studies revealed that FHL2 can act as a transcriptional co-regulator to regulate gene transcription in the cell/tissue specific pattern [7, 26]. By employing co-immunoprecipitation and chromatin immunoprecipitation methods, our study indicated that FHL2 may form complexes with AP-1 and NF-κB to regulate *Egf* and *Egfr* transcription in GCs, respectively. Besides, FHL2 deficiency inhibited EGF-induced GC growth, attenuated *ErbB* signaling and its downstream pathways such as PI3K/AKT. These indicate that FHL2 regulates follicular development by interacting with EGF/EGFR signaling.

Gonadotropins, including FSH and LH, via binding with G protein-coupled receptors (GPCR), play key roles in regulating female follicular development and ovulation. Crosstalk of GPCRs with tyrosine kinase receptor (RTKs) are intrinsically linked to follicular development and ovulation [16, 19]. A number of studies, including ours, demonstrated that EGFR signaling was transactivated and involved both in FSH stimulated follicular development and LH triggered ovulation [14, 37]. The ovulatory LH surge simultaneously leads to upregulation of EGF signaling in GCs, which is then transmitted to the cumulus cells and oocytes, leading to GC luteinization, meiotic resumption and ovulation [38]. In the current study, we demonstrated that FHL2 interacted with EGF/EGFR signaling, implying EGFR signaling might be a functional bridge for FHL2 between GPCR signaling pathway during follicular development and ovulation.

EGF, serves as an upstream autocrine/paracrine growth factor, regulating GC proliferation and differentiation [38]. EGF also serves as an important upstream regulator of Hippo/YAP pathway. We confirmed here that EGF treatment activates PI3K/AKT and MAPK/ERK signaling pathways and activates YAP1 in GCs. Intriguingly, we found that EGF treatment also induced FHL2 upregulation and the nuclear accumulation, indicating that EGF was also an upstream regulator of FHL2. Together with the finding that FHL2 deficiency inhibited the ability of EGF to induce itself and EGFR expression, these data indicate that FHL2 is involved in EGF/EGFR/YAP1 autocrine/paracrine loop.

It is worthy to notice that FHL2 was upregulated in human GCs or ovarian tissues of polycystic ovary syndrome (PCOS) patients [39]. However, FHL2 overexpression via intrabursal lentivirus injection only induced polycystic ovarian morphology (PCOM, a mild PCOS features) in a rat model. Female rats injected with FHL2 overexpression lentivirus showed more preantral follicles and small antral follicles in ovaries compared with WT, which is partially consistent with our finding that FHL2 deletion deceased numbers of growing follicles. Besides, ovulation was not totally blocked in the FHL2 overexpression rat model, a finding supported by the presence of corpora lutea in the FHL2 overexpressed rats ovary [39]. The authors suggest that the discrepancies between FHL2-overexpressing rat ovary phenotype and that of PCOS patients may be explained by partial involvement of FHL2 in the ovarian features of PCOS. Considering FHL2 overexpression induced PCOM or ovarian carcinogenesis [7, 24, 39], and FHL2 deficiency decreased follicular development and fertility, it seems that FHL2 may play bidirectional roles in ovarian physiology. Balanced FHL2 expression level is critical to maintain the normal ovarian physiology and female fertility.

**Fig. 7:**
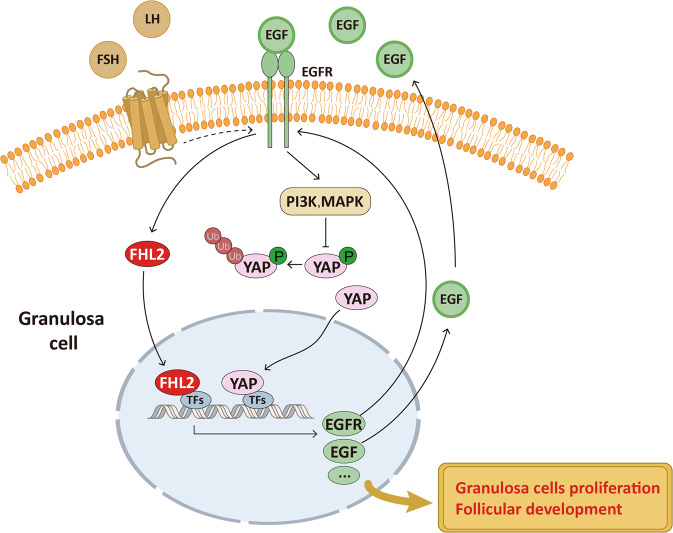
A schematic diagram showing the proposed mechanism for FHL2 to regulate GC proliferation and follicular development.

In conclusion, our present study demonstrated that FHL2 deficiency inhibited GCs proliferation, impaired female mice follicular development and fertility. FHL2 depletion also impaired EGFR and Hippo/YAP signaling in GCs. Mechanistically, FHL2 regulated *Egf* and *Egfr* transcription via binding with transcriptional factors. EGF in turn, not only induced FHL2 expression and nuclear accumulation, but also stimulated YAP1 activation and expression. Collectively, our study provide evidence that FHL2, via interacting with EGF/EGFR and Hippo/YAP signaling pathways, play an important role in regulating follicular development and female fertility (Fig. 7). Balanced FHL2 expression level is required to maintain ovarian physiology and fertility.

## Experimental procedures

### Animals and ethics statement

C57BL/6 J were bred in the experimental animal center of Huazhong Agricultural University. All studies were approved by the Huazhong Agricultural University Animal Care and Use Committee (Permit Number HZAUMO-2015-019). All mice were housed at an ambient temperature of 21 ± 2 °C, and given free access to food and water under a 12 h light/dark cycle. Age-matched female mice were randomly assigned for the following experiments.

### Construction of *Fhl2* knock-out mouse using CRISPR/Cas9 technology

According to the protocol for constructing gene-editing mice [40], the experimental procedure is as follows: (1) Four sgRNAs were designed by using sgRNAcas9 software [41] were shown in Supplementary Table S1. The oligonucleotides for sgRNAs were annealed and ligated into pUC57-sgRNA expression vector. (2) The amplified Cas9 PCR product was gel purified and used as the template for in vitro transcription (IVT) using mMESSAGE mMACHINE T7 ULTRA kit (Life Technologies, Gaithersburg, MD, USA). The four FHL2 pUC57-sgRNA plasmids were used as the template for IVT using a MEGAshortscript T7 kit (Life Technologies, Gaithersburg, MD, USA). Both the Cas9 mRNA and the sgRNAs were purified using a MEGAclear kit (Life Technologies, Gaithersburg, MD, USA) and eluted into RNase-free water. The qualities of the RNAs were checked by gel electrophoresis. (3) Fertilized embryos were collected and in vitro transcribed Cas9 mRNA (50 ng/ml) and sgRNA (20 ng/ml) were injected into the pronuclei or cytoplasm of fertilized embryos. To produce mutant mice, 1-cell stage embryos were transferred into the ampulla of the oviduct (9–18 embryos per oviduct) of pseudopregnant females. The *Fhl2* KO mice were constructed in a C57BL/6 J background by deleting an exon3 to exon5 genomic fragment generated by Nanjing Biomedical Research Institute of Nanjing University (NBRI, Nanjing, China). Genomic DNA was extracted from newborn mouse tissues using Genomic DNA kit (Tiangen, Beijing, China). PCR products spanning target sites of 453 bp (wild type) and 440 bp (*Fhl2* KO) were generated. PCR primers for WT and knock-out mice were shown in Supplementary Fig. S4B.

### Ovarian histology and serial follicle counting

Ovaries from *Fhl2* KO or WT mice were immediately fixed in freshly prepared 4% paraformaldehyde and processed for paraffin embedding with a protocol as described before [23]. The samples were dehydrated, and embedded in paraffin, and 5 µm serial sections were cut. Sections were placed in order onto positively charged glass slides followed by Hematoxylin and Eosin staining (HE). Follicles were counted in every 5th serial section. Only follicles containing a clearly stained oocyte nucleus were counted.

### Primary culture of GCs and transfection

GC culture was performed under the protocol designed by Luo with a slight modification [42]. The cell suspensions were collected and cultured in F12/DMEM (Gibco, Carlsbad, CA, USA) supplemented with 10% FBS (Gibco, Carlsbad, CA, USA) and 1% Penicillin–Streptomycin in 6-well plates at 37 °C in a CO_2_ incubator. After 48 h of culture, the medium was replaced to remove unattached cells and this procedure was repeated every 48 h. Mouse-specific scrambled siRNA and *Fhl2* specific siRNA were purchased from Dharmacon (Dharmacon, Lafayette, CO, USA). Lipofectamine RNAiMAX Transfection Reagent (Thermo Fisher Scientific, Waltham, MA) was used for siRNA transfection according to the manufacturer’s instructions. GCs were transfected with empty vector MXIV and FHL2 overexpression constructs (Genewiz, Suzhou, China) using Lipo 3000 (Thermo Fisher Scientific, Waltham, MA) according to the manufacturer’s instructions.

### Immunohistochemistry

A previously described peroxidase-based immunohistochemistry protocol was used to detect FHL2 expression in paraffin-embedded ovarian tissues [36]. Anti-FHL2 antibody (Proteintech, Wuhan, China) was used at 1:200. Subsequently, the sections were incubated with biotinylated secondary antibody (1:2000) (Boster Biotechnology, Wuhan, China) and avidin-biotin-peroxidase (Boster Biotechnology, Wuhan, China) before being exposed to diaminobenzidine (Servicebio, Wuhan, China, Wuhan, China) and counterstained with hematoxylin (Servicebio, Wuhan, China).

### Immunofluorescence and confocal microscopy

Briefly, GCs were seeded onto glass coverslips and incubated in a growth medium (10% FBS) until 60% confluent, and then treated with or without 20 ng/ml EGF for 48 h (in a medium containing 1% FBS). Next, cells were fixed in freshly prepared 4% paraformaldehyde for 30 min before blocking in PBS containing 5% BSA and 0.05% Triton-100 for 1 h at room temperature. Coverslips were incubated with primary antibodies overnight at 4 °C, followed by incubation with the secondary antibodies for 2 h at room temperature. Nuclei were labeled in 1 μg/ml of DAPI, and cells were examined with a confocal laser scanning microscope (Zeiss LSM 510 META, Carl Zeiss Imaging, Germany).

### RNA-sequencing library construction and data processing

GC total RNA was extracted using TRIzol (Invitrogen, CA, USA) and subjected to DNAase treatment using the RNase-Free DNase Set (Qiagen, Hilden, Germany) according to the manufacturer’s instructions. 2 μg total RNAs were used for stranded RNA sequencing library preparation using KC^TM^ Stranded mRNA Library Prep Kit for Illumina® (Seqhealth, Wuhan, China). Libraries were sequenced on the Novaseq 6000 sequencer (Illumina) with the PE150 model. Raw paired-end sequencing reads were aligned to mice GRCm38/mm10 reference genome by HISAT2 (2.1.0). FPKMs (fragments per kilobase of transcript per million mapped reads) were calculated using Cufflinks (v2.1.1). The genes with *q* ≤ 0.05 were identified as the differentially expressed genes. GO and KEGG analyses were performed using Clusterprofile. GSEA was analyzed by GSEA 4.1.0.

### Western Blot

GCs were washed in cold PBS and lysed with RIPA Lysis Buffer (Servicebio, Wuhan, China) containing protease inhibitor cocktail (Servicebio, Wuhan, China). Lysates were centrifuged at 12,000 × *g* for 15 min at 4 °C, to obtain the total cell lysates. Western Blot analysis was performed using standard protocols. Antibodies used are listed in Supplementary Table S2.

### QRT–PCR analysis of gene expression

Total RNA was isolated from primary cultures of GCs using TRIzol reagent (Invitrogen, CA, USA) according to the manufacturer’s instructions. The first-strand cDNA was synthesized from 1 μg total RNA and reverse transcription was completed using a SuperScript III First-Strand Synthesis System (Life Technlogy, Grand Island, USA). QRT-PCR was conducted with the QuantiTect SYBR® Green PCR Kit (Qiagen, Dusseldorf, Germany) on Bio-Rad instrument. The 2^−ΔΔCT^ method was used for data analysis. The sequences of primers for qRT-PCR were shown in Supplementary Table S1.

### Cell viability analysis

Cell Counting Kit-8 (CCK-8) was employed to determine whether FHL2 and EGF were associated with changes in cell viability. Granulosa cells were transfected with control vectors or FHL2-expressing vectors, or non-targeting control siRNA or FHL2 siRNA for 72 h, and/or treated with EGF for 48 h before adding the CCK-8 buffer using a protocol described by the manufacturer (Dojindo Molecular, Kumamoto, Japan).

### Cell cycle and apoptosis analysis

Cell cycle and apoptosis analysis were performed by flow cytometry. Control and treated cells were trypsinized, fixed and permeabilized with 75% ethanol in Ca^2^^+^/Mg^2+^ free PBS, and stored at −20 °C. The cells were then labeled with propidium iodide for 30 min at 37 °C and flow cytometry was performed to determine the cell-cycle distribution. Apoptosis was analyzed by cell surface presence of Annexin V using the Annexin V-APC/PI Dual Staining Apoptosis Assay Kit as described by the manufacturer (KeyGEN BioTECH, Nanjing, China).

### EGF ELISA assay

The culture media of granulosa cells transfected with control vectors or FHL2-expressing vectors, or non-targeting control siRNA or FHL2 siRNA were collected. The amount of EGF was examined by an EGF ELISA kit according to the manufactory instructions (Yuanye, Shanghai, China).

### Co-immunoprecipitation assays and Chromatin immunoprecipitation Assay

The cell lysates were incubated overnight with rabbit antibodies against FHL2, or normal rabbit IgG. Twenty microliters of protein A/G agarose beads were added to the mixture and incubated for 30 min at 4 °C. The beads were then pelleted by centrifugation and washed five times with cell lysis buffer. The pull-down proteins were fragmented by western blot using a protocol described before [7] and probed with an anti-NF-κB and anti-AP-1 antibody. An aliquot of total cell lysate was used as input. Chromatin immunoprecipitation was performed using an EZ Chromatin Immunoprecipitation Assay Kit with a protocol described by the manufacturer (EMD Millipore, Billerica, MA, USA). Pre-cleared chromatin was immunoprecipitated with rabbit polyclonal antibodies against FHL2, normal rabbit IgG or antibody against acetyl Histone H3 (included in the kit). The total cell chromatin extract was used as input. Precipitated chromatin was amplified with Taq DNA polymerase (Aidlab Biotechnologies, Beijing, China). The primer pairs are shown in Supplementary Table S1.

## Statistical analysis

Statistical analysis was performed using GraphPad Prism software (GraphPad Software, Inc., La Jolla, CA, USA). Data were analyzed for the significance of difference by one-way ANOVA (multiple groups) or Student’s *t*-test (two groups). A value of *P* < 0.05 was considered significant.

## Supplementary information

Supplementary tables

supplementary figure summary

Figure S1

Figure S2

Figure S3

Figure S4

Figure S5

Figure S6

Figure S7

Figure S8

Figure S9

Figure S10

aj-checklist-

## Data Availability

The data underlying this article will be shared on reasonable request to the corresponding author.
