# Supplementary material for: FHL2 deficiency impairs follicular development and fertility by attenuating EGF/EGFR/YAP signaling in ovarian granulosa cells
Source: Cell Death Dis. 2023 Apr 5;14(4):239. doi: 10.1038/s41419-023-05759-3 (PMC10073124; doi:10.1038/s41419-023-05759-3)
Supplement: Supplementary file 1 — Supplementary tables [file 41419_2023_5759_MOESM1_ESM.docx]

Supplemental Table 1. Primer information for SgRNA, qRT-PCR, and ChIP-PCR

| Prime name | Primer Sequence |
| --- | --- |
| sgRNA1 | GCTGGCTTGTCACTTCCGCGTGG |
| sgRNA2 | GAGACCAACTTACTATGGGCTGG |
| sgRNA3 | TCTGCATCACCAGGCTTAAGGGG |
| sgRNA4 | GCCTGGTGATGCAGAACCATTGG |
| *Egf*-F(QRT-PCR) | TTCTCACAAGGAAAGAGCATCTC |
| *Egf* -R(QRT-PCR) | GTCCTGTCCCGTTAAGGAAAAC |
| *Egfr*-F(QRT-PCR) | CCAACTATGGGACAAACAGAA |
| *Egfr*-R(QRT-PCR) | ATCGCACAGCACCAATCA |
| *Pcna*-F(QRT-PCR) | TAAAGAAGAGGAGGCGGTAA |
| *Pcna* -R(QRT-PCR) | TAAGTGTCCCATGTCAGCAA |
| *Bax*-F(QRT-PCR) | CAGGATGCGTCCACCAAGAA |
| *Bax*-R(QRT-PCR) | GCAAAGTAGAAGAGGGCAACCA |
| *Bcl2*-F(QRT-PCR) | TGGAGAGCGTCAACAGGGAGA |
| *Bcl2*-R(QRT-PCR) | GCCAGGAGAAATCAAACAGAGGT |
| *Caspase-3*-F(QRT-PCR) | AGCAGCTTTGTGTGTGTGATTCTAA |
| *Caspase-3*-R(QRT-PCR) | AGTTTCGGCTTTCCAGTCAGAC |
| *Fhl2*-F (QRT-PCR) | GGCAACGCTTCACAGCACGG |
| *Fhl2*-R (QRT-PCR) | GACAGGGAGCACTTCTTACA |
| *β-actin*-F (QRT-PCR) | GATTACTGCTCTGGCTCCTAGC |
| *β-actin*-R (QRT-PCR) | GACTCATCGTACTCCTGCTTGC |
| *Egf*-F (ChIP -PCR) | GTGGGATAGCTCAGTTTG |
| *Egf* -R (ChIP-PCR) | GTCACCTGCACTAATGTC |
| *Egfr*-F (ChIP-PCR) | ATCTACCATTAGTCGATGTC |
| *Egfr*-R (ChIP-PCR) | AGTAGAGCGGGGTTTC |

Supplemental Table 2. Antibodies information used for western blot and immunostaining

| **Antibody name** | **Dilution** | **Supplier** | **Cat. No** |
| --- | --- | --- | --- |
| FHL2 | 1:700 | Proteintech | 21619-AP |
| FHL2 (Co-IP) | 1:20 | Abmart | TD13015s |
| PCNA | 1:1000 | Proteintech | 10205-2-AP |
| Cleaved Caspase-3 | 1:1000 | Ruiying Biological | RLC0006 |
| phospho-P44/42 MAPK (T202/Y204) | 1:1000 | Cell Signaling | 4370 |
| Phospho-AKT (Ser473) | 1:1000 | Cell Signaling | 9271 |
| Bcl2 | 1:1000 | Proteintech | 26593-1-AP |
| FOXO1 | 1:1000 | Cell Signaling | 2880 |
| p-YAP（Ser127） | 1:1000 | Cell Signaling | 4911 |
| AKT | 1:1000 | Cell Signaling | 4691 |
| c-Fos (Co-IP) | 1:1000 | Proteintech | 66590-1-Ig |
| NF-κBp65 (Co-IP) | 1:1000 | Abmart | T55034s |
| Bax | 1:1000 | Cell Signaling | 14796 |
| GAPDH | 1:10000 | Proteintech | 10494-1-AP |
| β-actin | 1:10000 | Affinity Biosciences | AF7018 |
| HRP Goat Anti-Mouse IgG (H+L) | 1:5300 | ABclonal | AS003 |
| HRP Goat Anti-Rabbit IgG (H+L) | 1:5300 | Proteintech | SA00001-2 |
| VeriBlot IP Detection Reagent (HRP)(Co-IP) | 1:500 | Abcam | ab131366 |
