# Supplementary material for: FHL2 deficiency impairs follicular development and fertility by attenuating EGF/EGFR/YAP signaling in ovarian granulosa cells
Source: Cell Death Dis. 2023 Apr 5;14(4):239. doi: 10.1038/s41419-023-05759-3 (PMC10073124; doi:10.1038/s41419-023-05759-3)
Supplement: Supplementary file 2 — supplementary figure summary [file 41419_2023_5759_MOESM2_ESM.docx]

Fig S1. FHL2 immunosignals in mice ovary.

Fig S2. Subcellular location of FHL2 in GCs

Fig S3. Subcellular expression of FHL2 in GCs

Fig S4. Construction and identification of *Fhl2* knockout mice

Fig S5. Ovaries size age-matched *Fhl2* KO and WT mice

Fig S6. Representative image showing the hematoxylin-eosin (HE) stained ovaries.

Fig S7. Follicle morphology at various developmental stages

Fig S8. Gene Set Enrichment Analysis (GSEA) in *Fhl2* KO and WT GCs

Fig S9. Relative western blotting quantification data of protein expressions

Fig S10. Immunosignals of cleaved-caspase 3 protein in *Fhl2* KO and WT mouse ovary.
