# Supplementary figures and images for: FHL2 deficiency impairs follicular development and fertility by attenuating EGF/EGFR/YAP signaling in ovarian granulosa cells
Source: Cell Death Dis. 2023 Apr 5;14(4):239. doi: 10.1038/s41419-023-05759-3 (PMC10073124; doi:10.1038/s41419-023-05759-3)

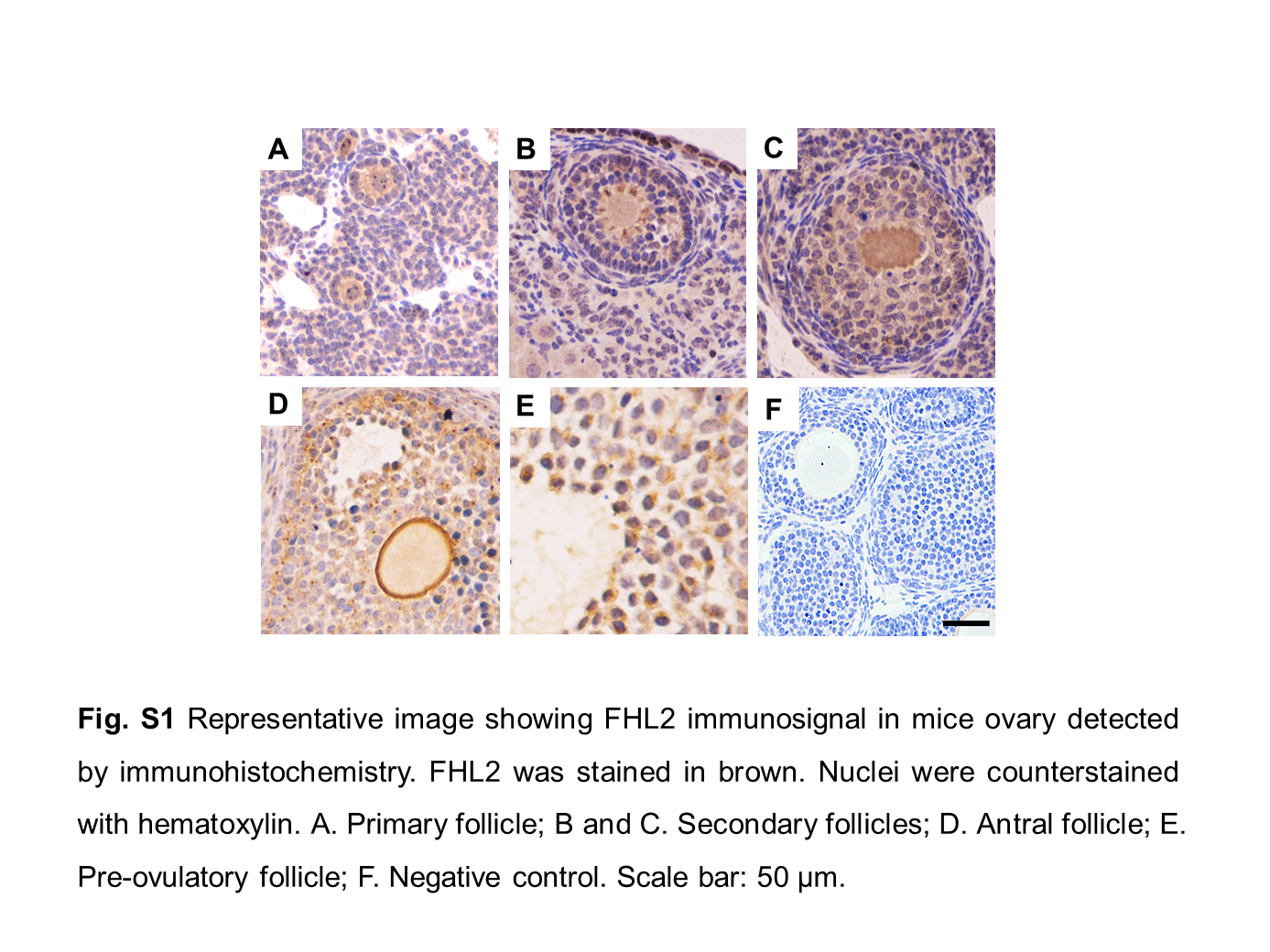

Supplement: Supplementary file 3 — Figure S1 [file 41419_2023_5759_MOESM3_ESM.png]

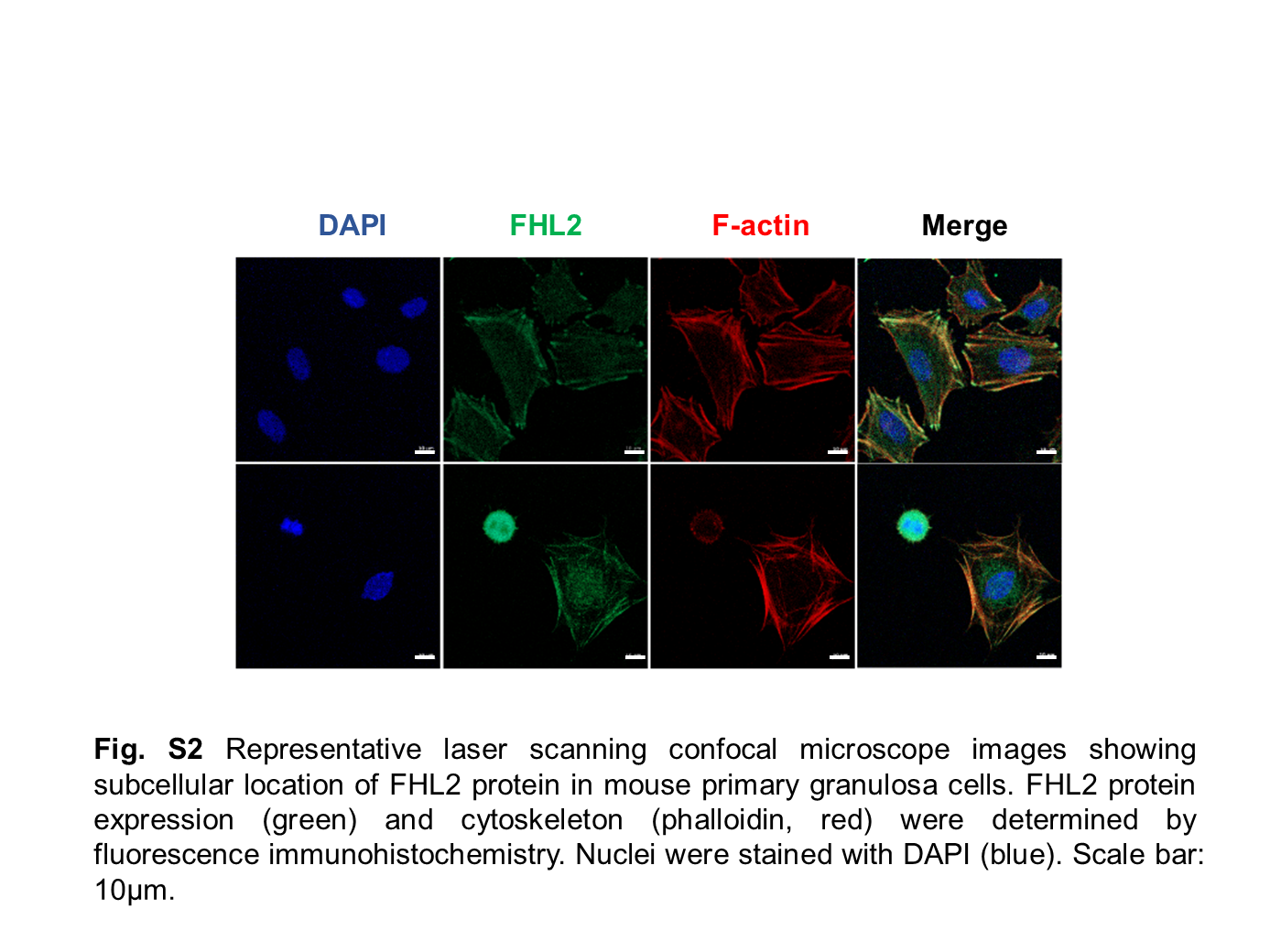

Supplement: Supplementary file 4 — Figure S2 [file 41419_2023_5759_MOESM4_ESM.png]

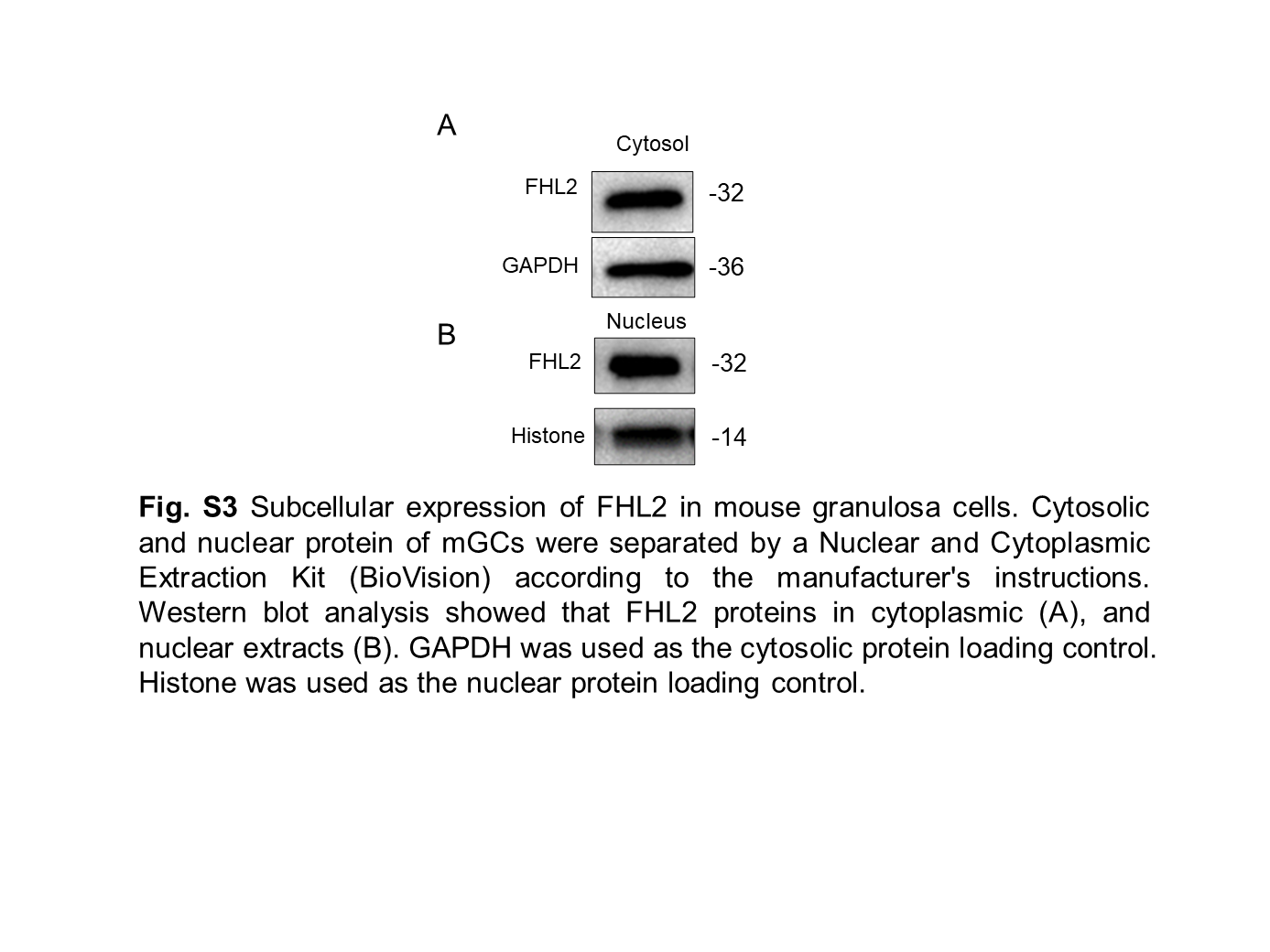

Supplement: Supplementary file 5 — Figure S3 [file 41419_2023_5759_MOESM5_ESM.png]

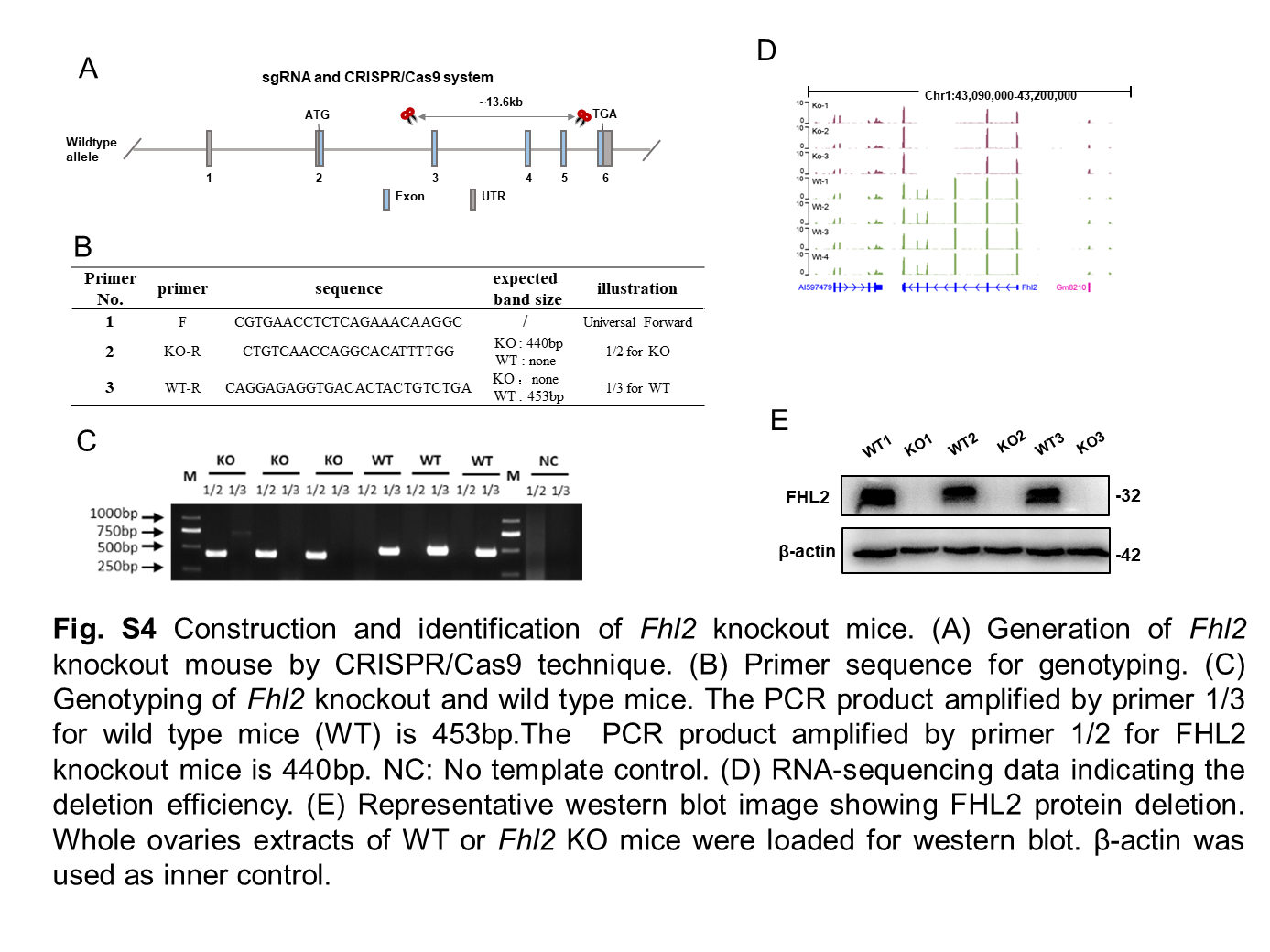

Supplement: Supplementary file 6 — Figure S4 [file 41419_2023_5759_MOESM6_ESM.png]

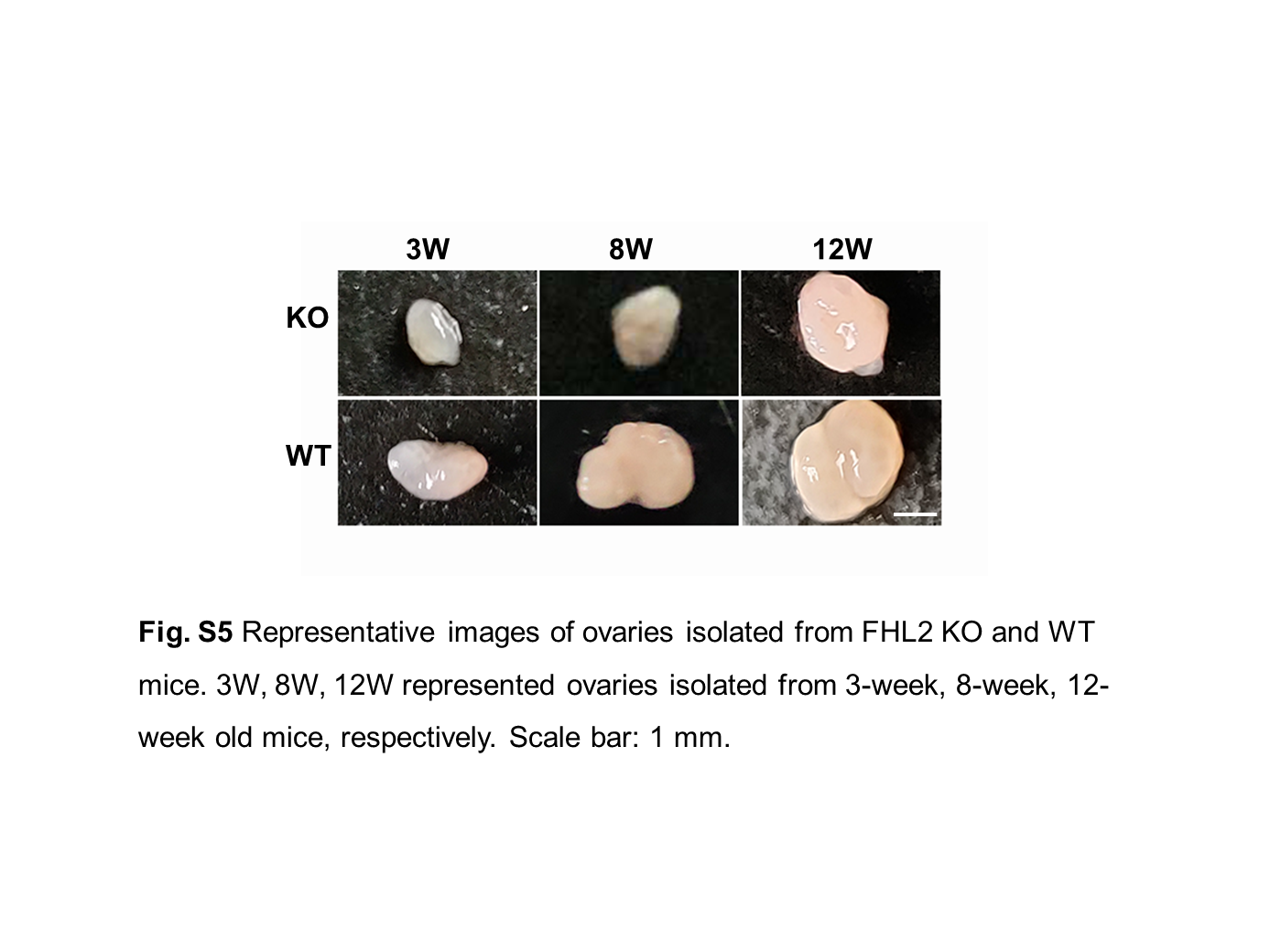

Supplement: Supplementary file 7 — Figure S5 [file 41419_2023_5759_MOESM7_ESM.png]

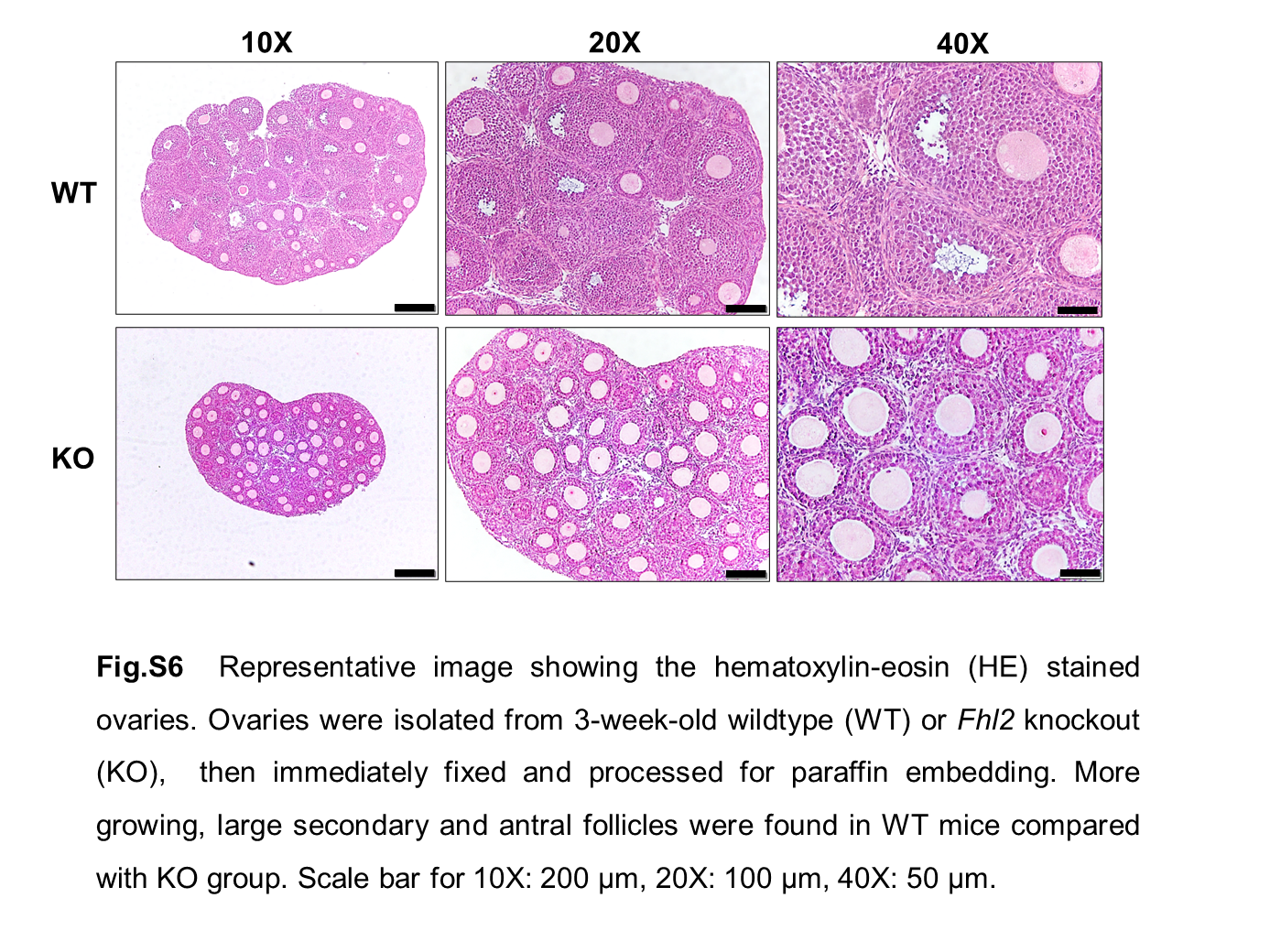

Supplement: Supplementary file 8 — Figure S6 [file 41419_2023_5759_MOESM8_ESM.png]

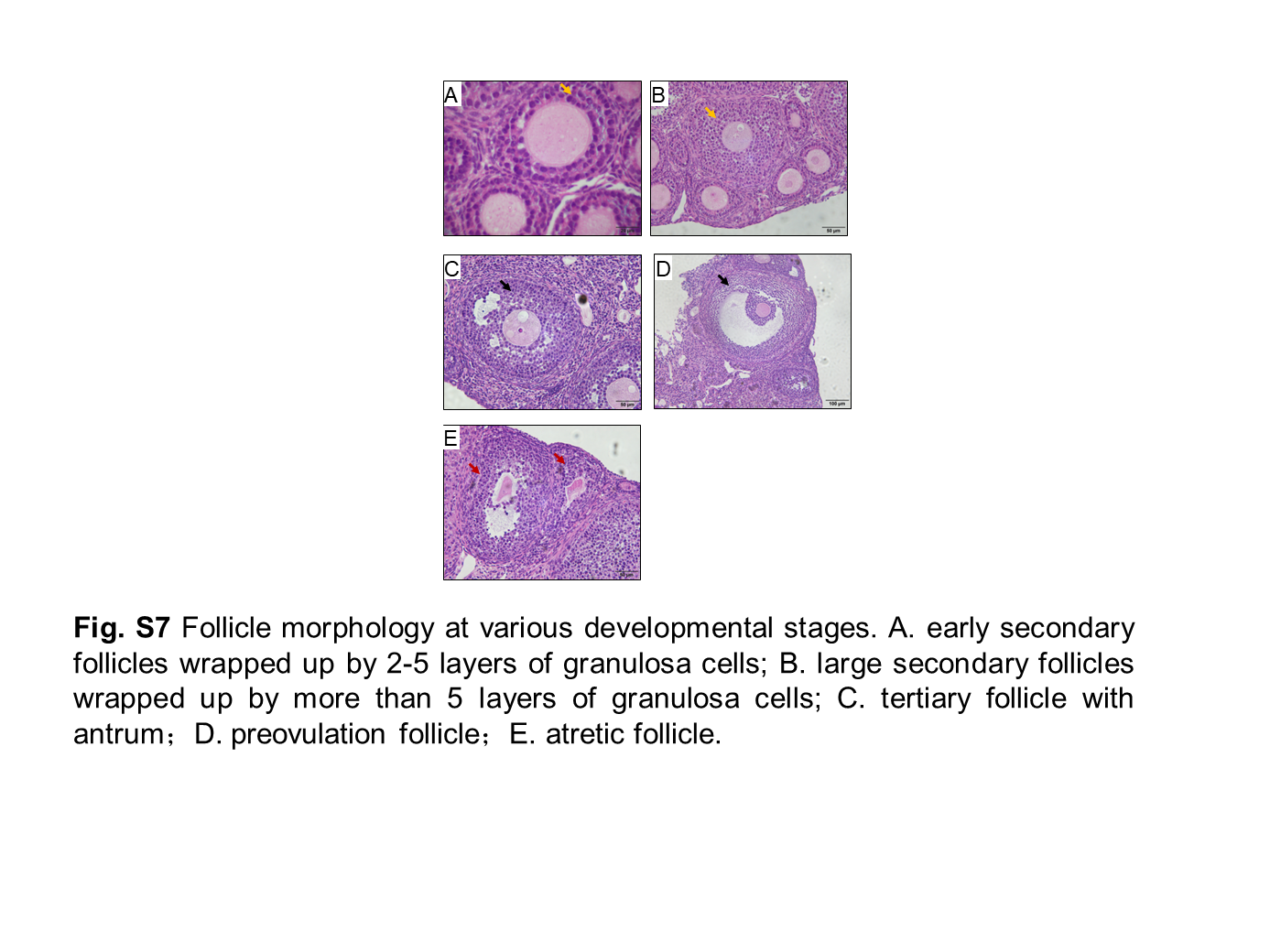

Supplement: Supplementary file 9 — Figure S7 [file 41419_2023_5759_MOESM9_ESM.png]

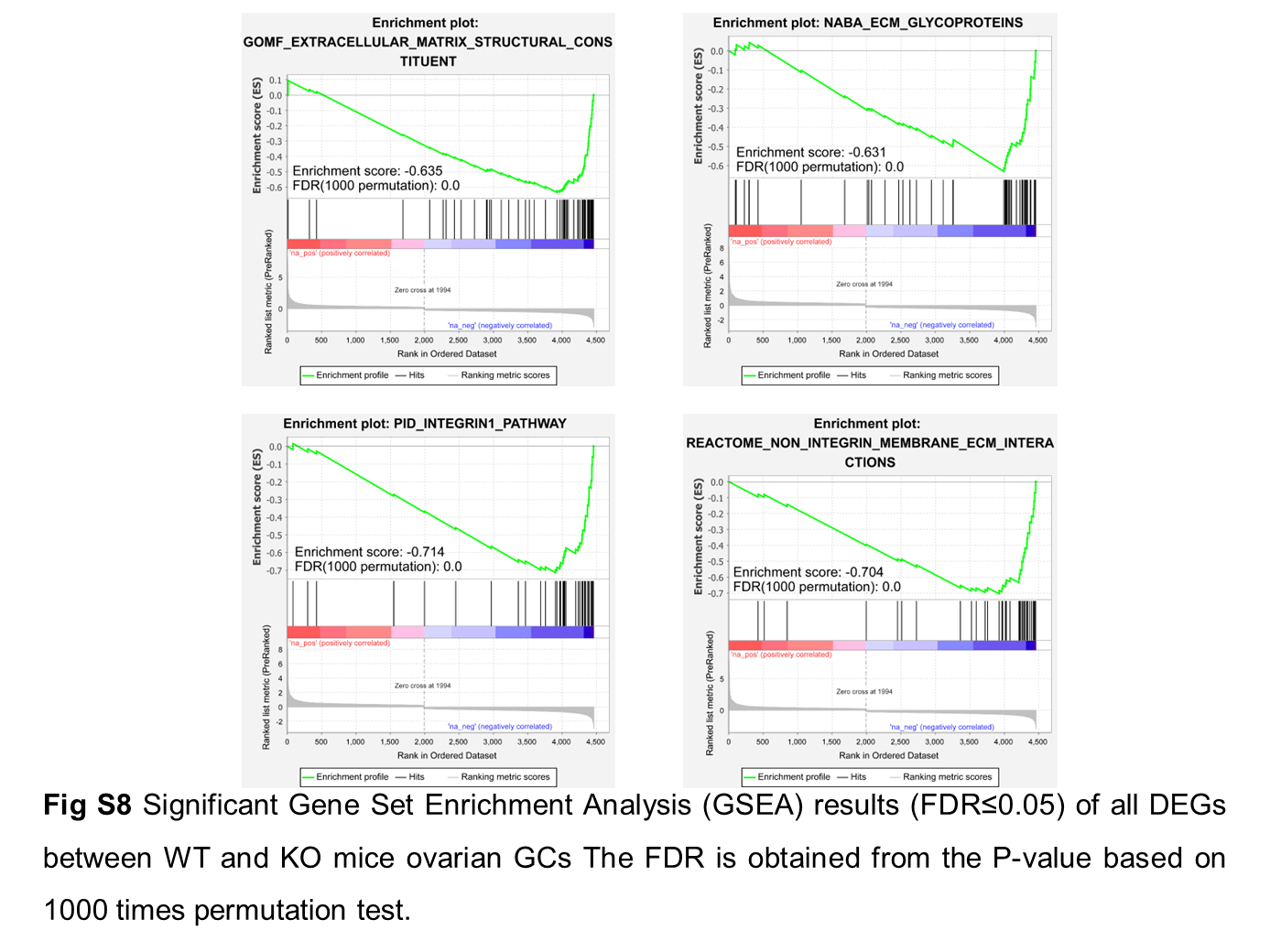

Supplement: Supplementary file 10 — Figure S8 [file 41419_2023_5759_MOESM10_ESM.png]

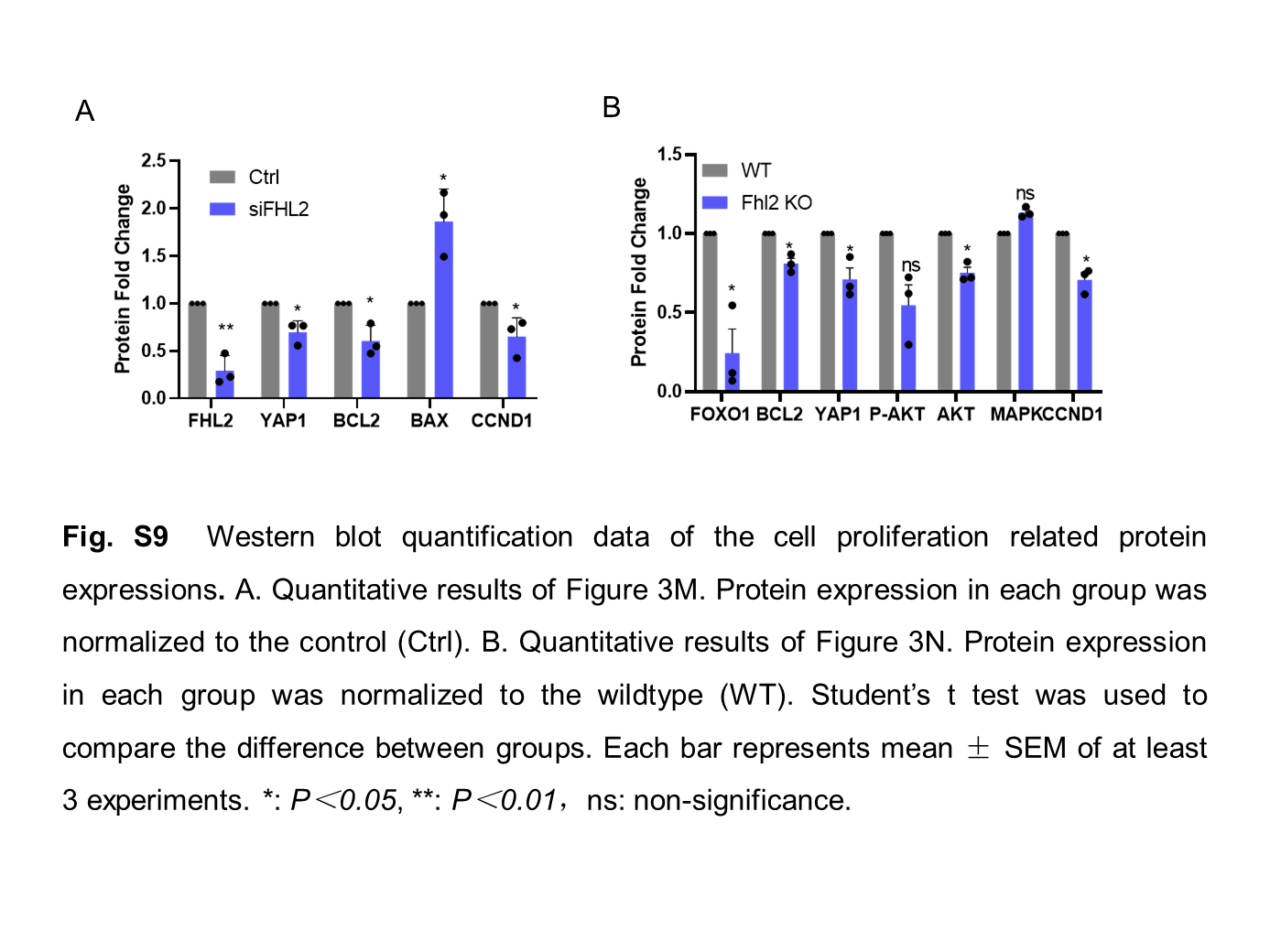

Supplement: Supplementary file 11 — Figure S9 [file 41419_2023_5759_MOESM11_ESM.png]

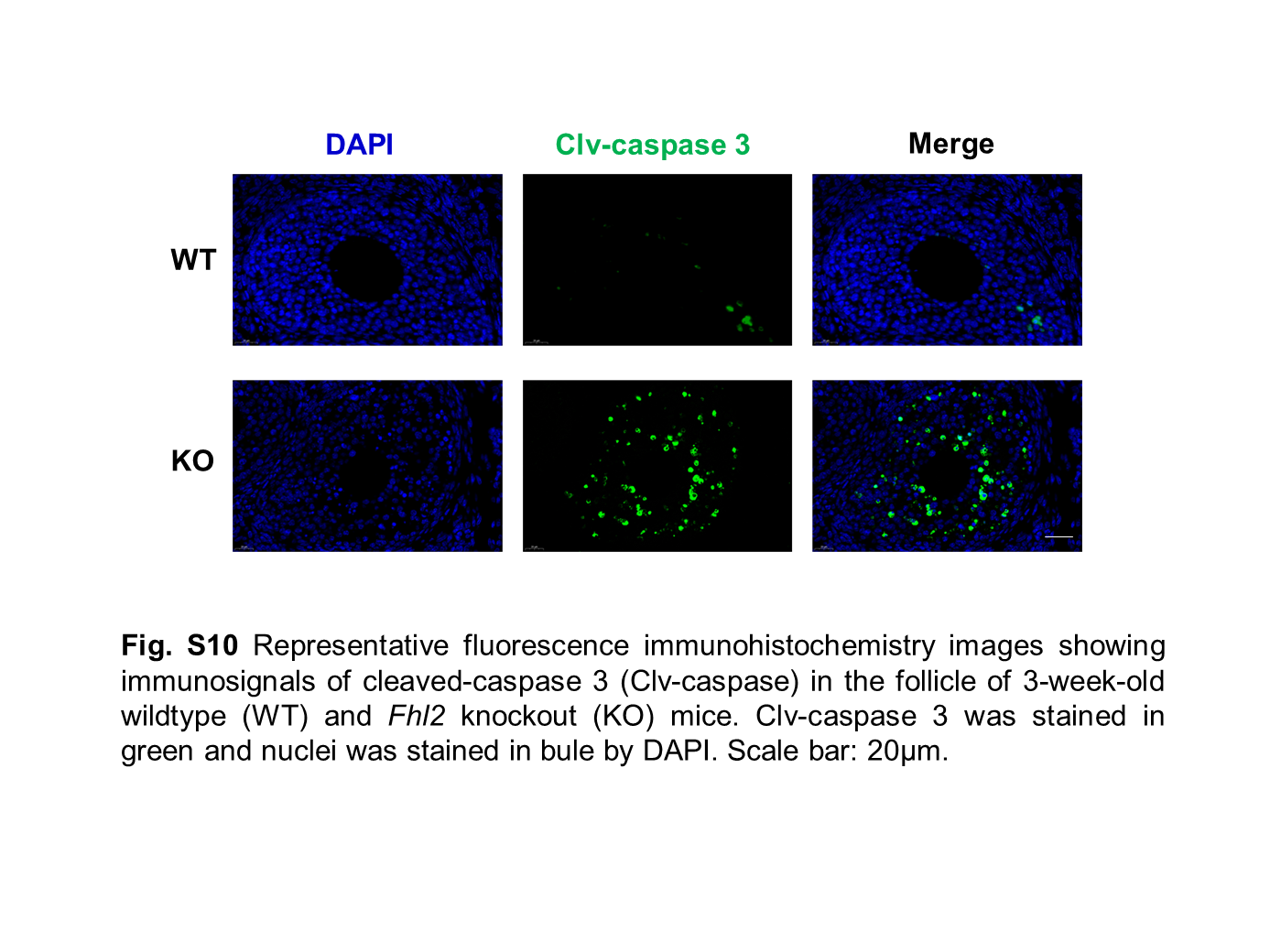

Supplement: Supplementary file 12 — Figure S10 [file 41419_2023_5759_MOESM12_ESM.png]
